# Embryonic Development of Avian Pineal Secretory Activity—A Lesson from the Goose Pineal Organs in Superfusion Culture

**DOI:** 10.3390/molecules26216329

**Published:** 2021-10-20

**Authors:** Maria Hanuszewska-Dominiak, Kamila Martyniuk, Bogdan Lewczuk

**Affiliations:** Department of Histology and Embryology, Faculty of Veterinary Medicine, University of Warmia and Mazury in Olsztyn, Oczapowskiego 13, 10-719 Olsztyn, Poland; maria.hanuszewska@uwm.edu.pl (M.H.-D.); kamila.kwiecinska@uwm.edu.pl (K.M.)

**Keywords:** pineal organ, melatonin, indoles, embryo, ontogeny, norepinephrine, birds

## Abstract

The embryonic ontogeny of pineal secretory activity in birds has been investigated almost exclusively in chickens. This study aimed to characterize this process in domestic geese. The pineal organs of embryos aged 18–28 days were incubated in superfusion culture under different light conditions for 4–5 days and treated with norepinephrine (NE). Melatonin (MLT) was measured by radioimmunoassay and other indoles by HPLC with fluorescence detection. Additionally, pineal organs were collected from embryos at 14–28 days of age and used to measure catecholamines by HPLC with electrochemical detection. MLT secretion increased with embryo age, most intensively between the 22nd and 24th days of life. The daily changes in MLT secretion under the 12 L:12D cycle occurred on the first day of culture, starting from an embryonic age of 24 days. MLT secretion was controlled by the light-dark cycle in all age groups studied. However, exposure to light during the scotophase did not alter the secretion of MLT. The endogenous oscillator expressed its activity in regulating MLT secretion in the pineal organs of embryos aged 24 days and older but could not generate a rhythm after one cycle. The rhythm of 5-hydroxytryptophan release during the first day of culture was found in the pineal organs of all embryos, while the rhythmic release of *N*-acetylserotonin and 5-methoxyindole acetic acid started at the age of 24 days. The proportion of released indoles changed with embryo age. NE caused a decrease in MLT secretion and provoked an increase in serotonin release. Incubation of the pineal organs induced the development of MLT secretory machinery and its diurnal rhythmicity. The pineal content of catecholamines increased prominently at the end of embryonic development.

## 1. Introduction

The embryonic development of pineal secretory activity in birds has been investigated almost exclusively in chickens [1,2,3,4,5,6,7,8,9,10]. In our previous study [11], the contents of 5-hydroxyindoles and 5-methoxyindoles were measured in the pineal organs of goose embryos aged 14–28 days (ED 14–ED 28). The domestic goose was chosen as the object of the study largely because of the characteristic metabolic profile of indoles with a high 5-HT level [12], opposite to that in the chicken [13], but it seems to be much more common in birds [14,15]. The results showed that pineal indole metabolism develops in this species in three steps. The pineal organ produces serotonin (5-HT) during the first phase, but it lacks the ability of its acetylation into *N*-acetylserotonin (NAS). The pineal contents of 5-HT, 5-hydroxytryptophan (5-HTRP), and 5-hydroxyindole acetic acid (5-HIAA) show daily variations from ED 14, when eggs are incubated under a 12L:12D cycle. In the second phase (ED 16), 5-HT acetylation is turned on, and in the last phase, the methylation of 5-hydroxyindoles is turned on. Melatonin (MLT) and 5-methoxyindole acetic acid (5-MIAA) are detectable in the pineal organs from ED 18 and show significant daily variations in their contents from ED 20.

A recently published study [16] demonstrated that a daily cycle of light and dark phases is insufficient to establish a well-entrained daily rhythm of MLT secretion in the pineal organs of 12-week old geese in superfusion culture. Direct pineal photoreception plays a less important role in regulating MLT secretion in the domestic goose than in the chicken [17,18,19,20] and the domestic turkey [21,22]. These differences may reflect dissimilarities in the histological organization and ultrastructure of the pineal organs in these birds [23,24,25,26,27,28,29]. The experiments with the culture of the goose pineal organs in the presence of norepinephrine (NE) and the measurements of catecholamine metabolites in these glands in a daily cycle in vivo strongly point to adrenergic innervation as a key regulator of MLT secretion in the pineal organs of geese aged several weeks [16].

Given these data, we decided to study the regulation of MLT secretion in the goose embryonic pineal organs in superfusion culture. The first objective of the study was to characterize the secretion of MLT from the pineal organs of goose embryos aged 18–28 days, incubated under a 12L:12D cycle. This goal was important from a comparative point of view because the majority of data on MLT synthesis in the embryonic avian pineal organ were obtained in vitro [2,4,30,31,32,33], and it was difficult to compare our previous results obtained in vivo [11] with those of other species. The second objective was to study the effects of the reversed dark-light cycle, light exposure at night, continuous darkness, and norepinephrine on MLT secretion. Next, because the results obtained using radioimmunoassay in this study showed that MLT secretion largely increased in the course of culture, we considered it important to check the changes in the release of other indoles to clarify this phenomenon better. Therefore, the third objective was to measure the release of indoles other than MLT by HPLC. Such measurements were performed for the first time in studies of avian pineal organs. The fourth objective was to analyze the content of catecholamines and their metabolites in the pineal organs of goose embryos in vivo.

## 2. Results

### 2.1. In Vitro Study

#### 2.1.1. Experiment I

##### Melatonin Secretion from Embryonic Pineal Organs Incubated under Different Light Conditions and Treated with NE

**Figure 1:**
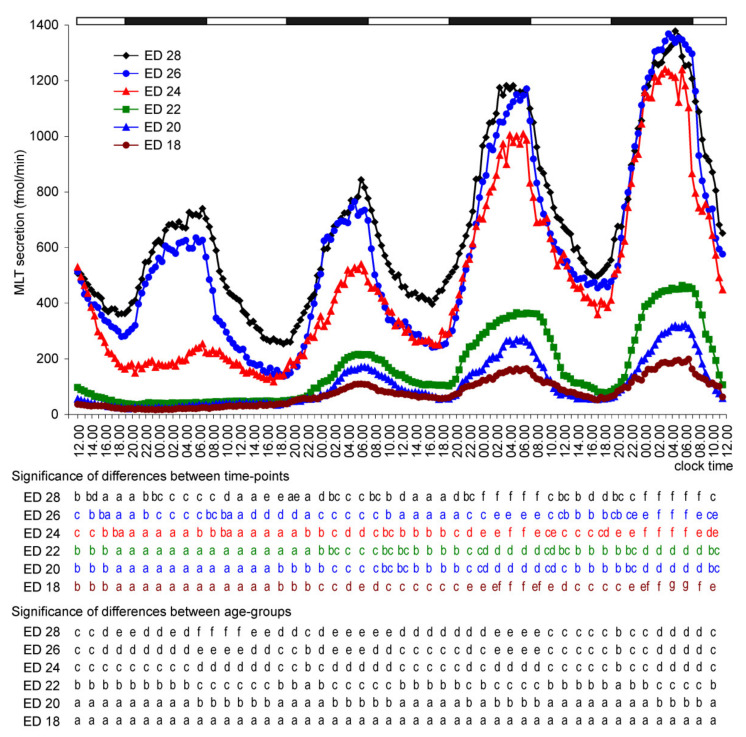
Secretion of MLT (means, *n* = 4) from pineal organs of goose embryos incubated under a 12L:12D cycle (photophase from 07.00 to 19.00). Color letters show differences between time-points within a group of explants prepared from embryos of the same age, and black letters show differences between these groups at the same time-point. The identical letters indicate means, which are not significantly different.

MLT secretion from the pineal organs of ED 18, ED 20, and ED 22 embryos slightly decreased in the first hours of culture. It then remained at a very low level (despite slow, successive, but statistically insignificant elevation) until the scotophase on the second day of incubation, when the first nocturnal peak occurred (Figure 1). On day 2, MLT secretion increased stepwise between 19.00 and 04.00 to reach the level approximately 3-fold (ED 18) to 5-fold (ED 20 and ED 22) higher than the previous daytime level, and next slowly decreased after 07.00. The night-time peaks of MLT secretion also occurred during the scotophases of days 3 and 4. These peaks were higher than the peak on day 2. MLT secretion levels during the photophases on days 3 and 4 were higher than those on days 1 and 2.

The decrease in MLT release in the initial phase of culture was more intense in the pineal organs of ED 24, ED 26, and ED 28 embryos than in the younger ones (Figure 1). In the pineal organ culture of ED 24 embryos, MLT secretion levels showed a small peak at the end of the first scotophase, being significantly higher between 5.30 and 6.30 than 18.00 and 22.00. Prominent nocturnal peaks of MLT secretion from these explants occurred during the following days of culture, and each peak was higher than the previous one. The daytime values also increased on consecutive culture days. The pineal organs of ED 26 and ED 28 embryos showed significant daily fluctuations in MLT secretion starting from the first day of culture (Figure 1). Similar to the case of ED 24 embryos, both daytime and night-time levels of MLT secretion increased in successive cycles. 

**Figure 2:**
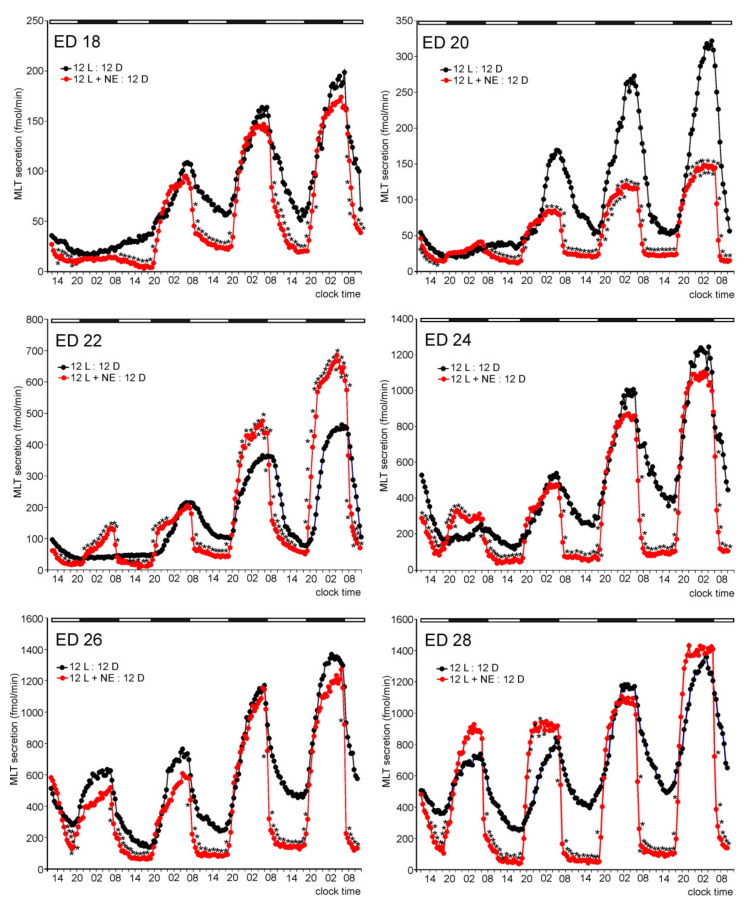
Comparison of MLT secretion (means, *n* = 4) from pineal organs of ED 18–ED 24 embryos incubated under a 12L:12D cycle with or without NE in the culture medium during photophase. Asterisks indicate significant differences between the pineal organs treated and untreated with NE.

The presence of NE in the culture medium during photophases prominently modified the daily fluctuations in MLT secretion from the embryonic pineal organs (Figure 2). The secretion of MLT was significantly lower during photophases in the NE-treated pineal organs than in the untreated ones starting from the first day of culture, independently of the age of embryos. The withdrawal of NE from the culture medium at the end of photophase resulted in a rapid increase in MLT secretion. The introduction of NE to the culture medium at the end of scotophase caused a rapid decline in secretion. The maximal levels of MLT secretion were mostly not affected by NE treatment, except for ED 20 and 22 embryos. In contrast to the untreated explants, a significant increase in MLT secretion was noted in the pineal organs of ED 20 and ED 22 embryos during the first day of culture. 

**Figure 3:**
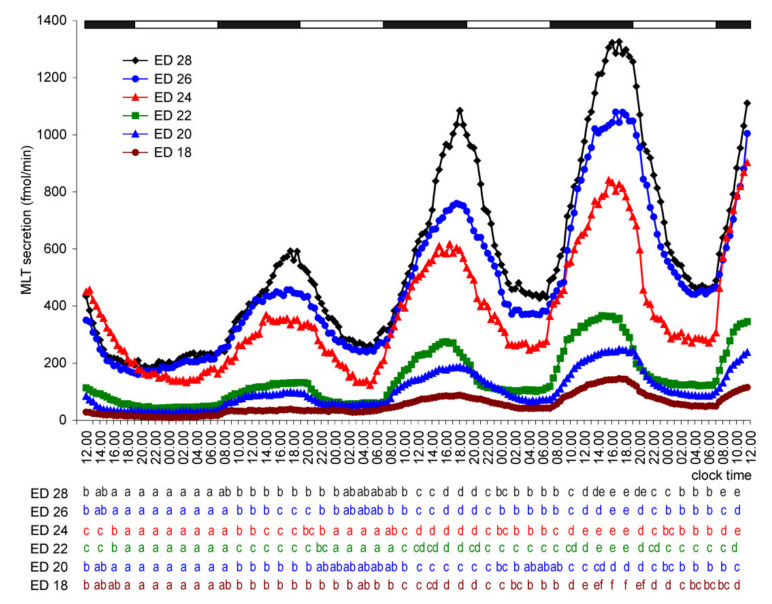
Secretion of MLT (means, *n* = 4) from pineal organs of goose embryos incubated under a reversed 12D:12L cycle (photophase from 19.00 to 07.00). Letters show differences between time-points within a group of explants prepared from embryos of the same age. The identical letters indicate means, which are not significantly different.

Incubation under a reversed, dark-light cycle (12D:12L) abolished the nocturnal peak of MLT secretion during the first day of culture (Figure 3). However, from day 2, the pineal organs of ED 20–ED 28 embryos secreted MLT in a reversed cycle, with peaks occurring between 07.00 and 19.00. MLT secretion from the pineal organs of ED 18 embryos increased significantly during the scotophase on day 2 but did not decrease during the following photophase. Cyclic secretion from these explants started on the third day of culture. Similar to the 12L:12D cycles, MLT secretion from the pineal organs increased on consecutive days of culture. 

**Figure 4:**
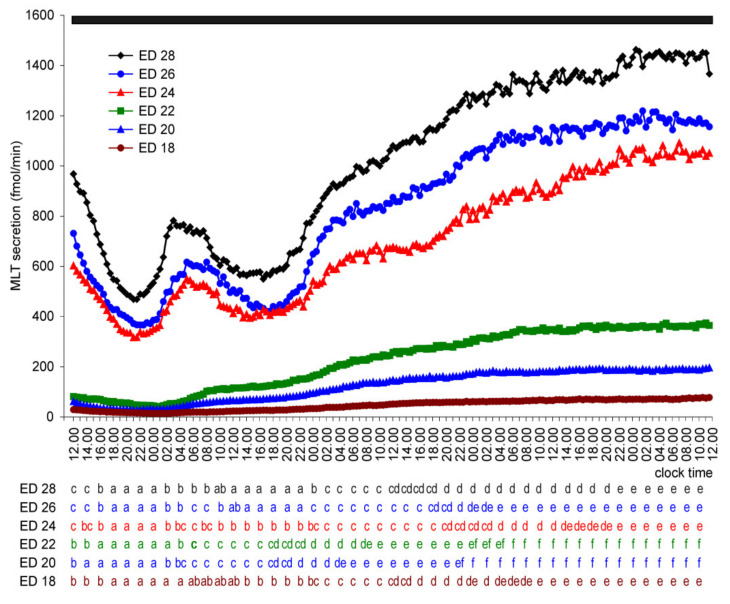
Secretion of MLT (means, *n* = 4) from pineal organs of goose embryos incubated under continuous darkness. Letters show differences between time-points within a group of explants prepared from embryos of the same age. The identical letters indicate means, which are not significantly different.

MLT secretion from the embryonic pineal organs incubated in continuous darkness decreased during the first hours of culture (Figure 4). Next, the secretion of MLT from the pineal organs of ED 18–ED 22 embryos increased stepwise and reached a plateau on the last day of culture. In contrast to these explants, MLT secretion from the pineal organs of ED 24–ED 28 embryos raised during the natural night on the first day of culture, remained elevated for 3–4 h, and then decreased during the natural day. Next, the secretion increased uninterrupted until it reached a stable level on the last day of culture.

##### Release of 5-HTRP, 5-HT, NAS, MLT, and 5-MIAA from Embryonic Pineal Organs Incubated under 12L:12D Cycle

**Figure 5:**
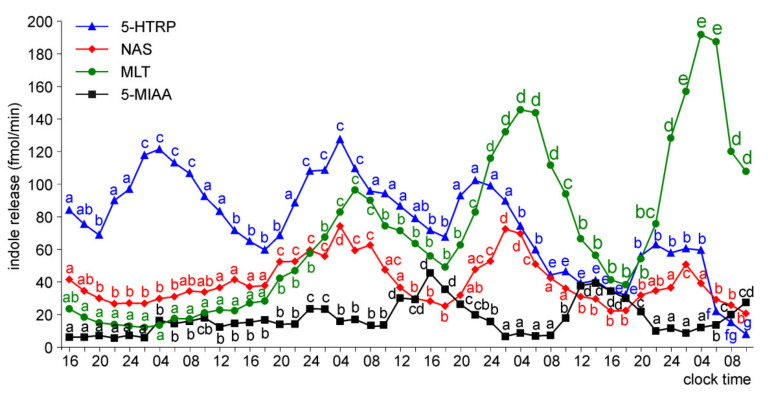
Release of 5-HTRP, NAS, MLT, and 5-MIAA (means, *n* = 3) from pineal organs of ED 18 embryos incubated under a 12L:12D cycle. The same letters indicate means, which are not significantly different between time-points.

The release of 5-HTRP by the pineal organs of ED 18 embryos showed significant daily fluctuations starting from the first day of culture, but the daily profiles and levels of 5-HTRP release changed during the experiment (Figure 5). During the first two days of culture, the 5-HTRP release increased stepwise in the first half of the scotophase and then slowly decreased between 04.00 and 18.00. On the third and fourth days of culture, the peaks were more variable in shape, and the release was lower than during the first two days. In contrast to 5-HTRP, daily rhythmicity did not occur in the release of NAS, MLT, and 5-MIAA on the first day of culture; however, it developed later. The release of NAS and MLT increased slowly during the first two days of culture (up to the end of the second scotophase) and then showed prominent daily changes with the highest levels in the late scotophase. Similarly, the release of MIAA increased during the first two days of culture and then showed significant daily changes; however, the peaks were in the second half of photophase. 

**Figure 6:**
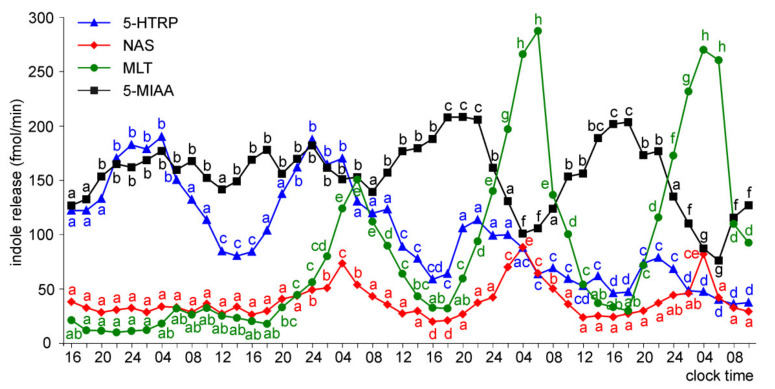
Release of 5-HTRP, NAS, MLT, and 5-MIAA (means, *n* = 3) from pineal organs of ED 20 embryos incubated under a 12L:12D cycle. The same letters indicate means, which are not significantly different between time-points.

The pineal organs of ED 20 embryos also released 5-HTRP in the daily rhythm during the entire culture period (Figure 6). The peaks of 5-HTRP release were significantly lower on the third and fourth days of culture than during the first two days. The NAS and MLT release showed significant daily fluctuations during the last three days of culture. The prominent rhythm of 5-MIAA release was noted during the second half of the culture. 

**Figure 7:**
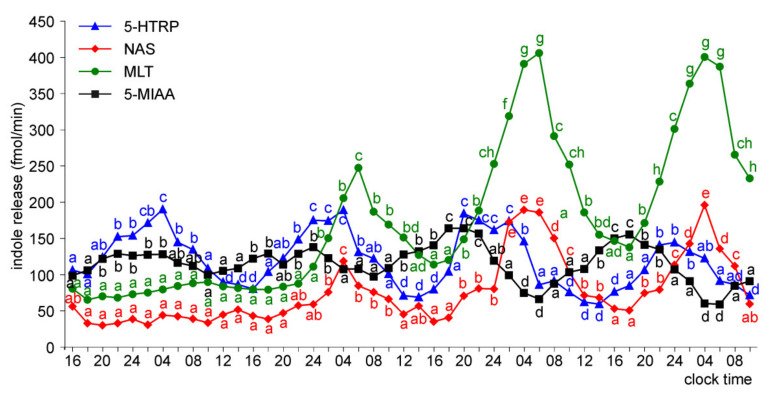
Release of 5-HTRP, NAS, MLT, and 5-MIAA (means, *n* = 3) from pineal organs of ED 22 embryos incubated under a 12L:12D cycle. The same letters indicate means, which are not significantly different between time-points.

The release of 5-HTRP from the pineal organs of ED 22 embryos showed significant daily changes throughout the experiment, with the peaks of similar values, except the last one, which was smaller (Figure 7). The night-time peaks of NAS and MLT occurred on the last three days of the experiment, and they were much higher on days 3 and 4 than on day 2. Similar to the ED 20 embryos, the release of 5-MIAA showed significant daily changes during the last two days of culture. The peaks of 5-MIAA release occurred during the transition between photophase and scotophase.

**Figure 8:**
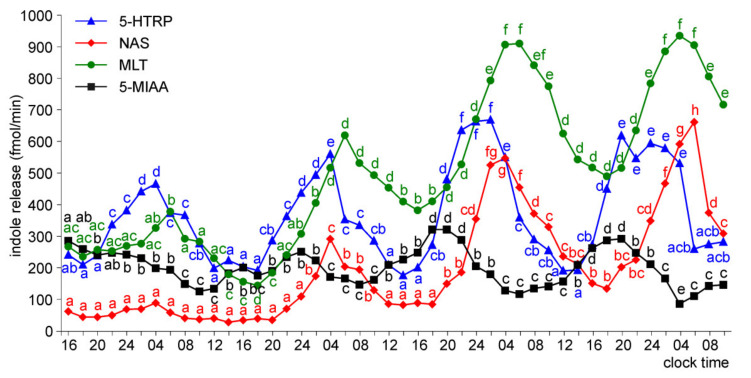
Release of 5-HTRP, NAS, MLT, and 5-MIAA (means, *n* = 3) from pineal organs of ED 24 embryos incubated under a 12L:12D cycle. The same letters indicate means, which are not significantly different between time-points.

In the superfusion culture of the pineal organs of ED 24 embryos, the prominent rhythm of 5-HTRP release occurred throughout the experiment (Figure 8). The release of NAS showed no significant changes on the first day of culture, while at the same time, the MLT secretion was significantly higher at 06.00 than 16.00 and 02.00. Both NAS and MLT showed prominent daily changes in their levels in the medium on the subsequent days of culture. Significant daily changes were also observed in the release of 5-MIAA. 

**Figure 9:**
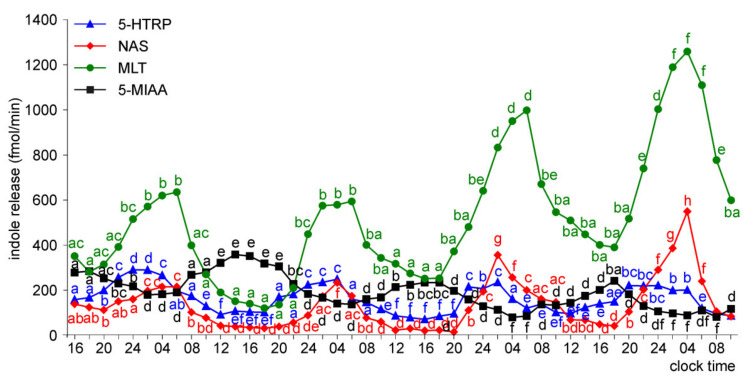
Release of 5-HTRP, NAS, MLT, and 5-MIAA (means, *n* = 3) from pineal organs of ED 26 embryos incubated under a 12L:12D cycle. The same letters indicate means, which are not significantly different between time-points.

**Figure 10:**
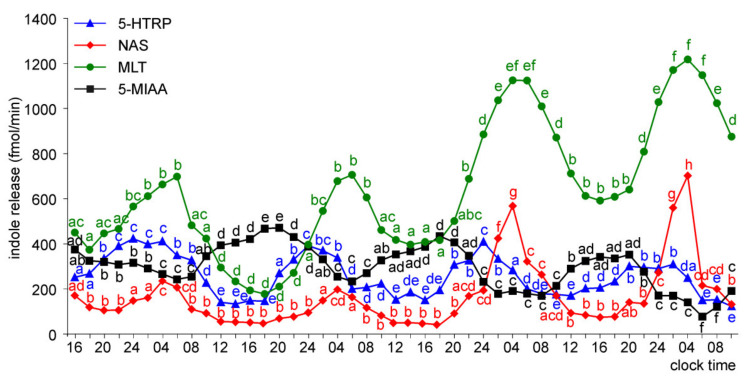
Release of 5-HTRP, NAS, MLT, and 5-MIAA (means, *n* = 3) from pineal organs of ED 28 embryos incubated under a 12L:12D cycle. The same letters indicate means, which are not significantly different between time-points.

The pineal organs of ED 26 and ED 28 embryos released 5-HTRP, NAS, MLT, and 5-MIAA in daily rhythm during the whole culture period (Figure 9 and Figure 10). The 5-HTRP, NAS, and MLT peaks occurred during the scotophase (peaks of 5-HTRP before NAS and MLT), and the peak of 5-MIAA occurred during the photophase and early scotophase. The maximal levels of NAS and MLT release increased during the course of culture, while the maximal levels of 5-HTRP and 5-MIAA slightly decreased. 

The concentration of 5-HT in the culture medium samples was below the quantification limit of the analytical method, independent of embryo age and incubation duration. 

The release of NAS, MLT, and 5-MIAA from the pineal organs increased with embryo age; however, this increase was not proportional to the embryo age and differed between compounds (Supplementary Materials, Figures S1–S4). During the first day of culture, the release of NAS was similar in the pineal organs of ED 18, ED 20, and ED 22 embryos. Compared to these explants, it was approximately 2-fold higher in the pineal organs of ED 24 embryos and approximately 5-fold higher in the pineal organs of ED 26 and ED 28 embryos. These changes were inconsistent with changes in MLT secretion. At the same time, the 5-MIAA release was approximately 10-fold higher in the pineal organs of ED 20 than in ED 18 embryos, while the differences between the pineal organs of ED 20 and ED 28 embryos did not reach 400%. In contrast to NAS, MLT, and 5-MIAA, the release of 5-HTRP from the pineal organs increased to ED 24 and then decreased. As a consequence, there were prominent differences in the ratio between the amounts of indoles released into the culture medium depending on embryo age. For example, the release of 5-HTRP on the first day of the experiment was much higher than the MLT release from the pineal organs of ED 18 and ED 20 embryos (Figure 5 and Figure 6); however, it was much lower than the MLT release from the organs of ED 26 and ED 28 embryos (Figure 9 and Figure 10). Similarly, the MLT to NAS release ratio altered with embryo age (Figure 5, Figure 6, Figure 7, Figure 8, Figure 9 and Figure 10).

The proportion of released indoles also changed during the course of culture. The ratio of 5-HTRP to MLT release decreased during the culture in all investigated embryos (except on day 2 in the pineal organs of ED 26 and ED 28 embryos), but this change was more prominent in the pineal organs of the younger embryos (Figure 5, Figure 6, Figure 7, Figure 8, Figure 9 and Figure 10). The ratio of MLT to NAS release increased during the culture of the pineal organs of ED 18 and ED 20 embryos (Figure 5 and Figure 6), remained almost unchanged in the case of ED 22 embryos (Figure 7), and decreased in the case of ED 24 embryos (Figure 8). In the culture of the pineal organs of ED 26 and ED 28 embryos, it was stable during the first half of culture and then increased (Figure 9 and Figure 10).

##### Release of 5-HTRP, 5-HT, NAS, MLT, and 5-MIAA from Embryonic Pineal Organs Incubated under 12 L:12D Cycle and Treated with NE during Photophases

**Figure 11:**
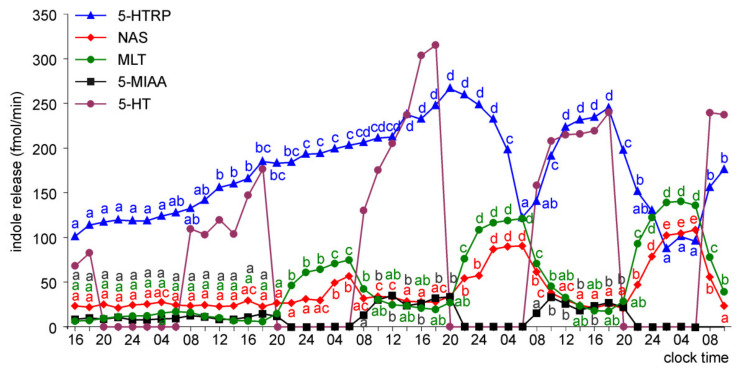
Release of 5-HTRP, NAS, MLT, and 5-MIAA (means, *n* = 3) from pineal organs of ED 18 embryos incubated under a 12L:12D cycle and treated with NE during photophase. The same letters indicate means, which are not significantly different between time-points.

Treatment with NE during the photophases largely modified the release of 5-hydroxyinoles and 5-methoxyindoles. In the superfusion culture of the pineal organs of ED 18 embryos, the release of NAS and MLT did not increase continuously during the first two days of incubation, like it did in untreated cultures. Therefore, the nocturnal peaks of NAS and MLT occurring on the second day of incubation had well-defined onset points (Figure 11). The presence of NE resulted in the immediate occurrence of a large increase in the release of 5-HT, and consequently, the NE-driven rhythm of 5-HT release. Treatment with NE disturbed the rhythmic release of 5-HTRP during the first half of the culture; however, daily fluctuations, with declines during scotophases, were present in the second half of the experiment. 5-MIAA was undetectable during scotophase on days 2, 3, and 4. 

**Figure 12:**
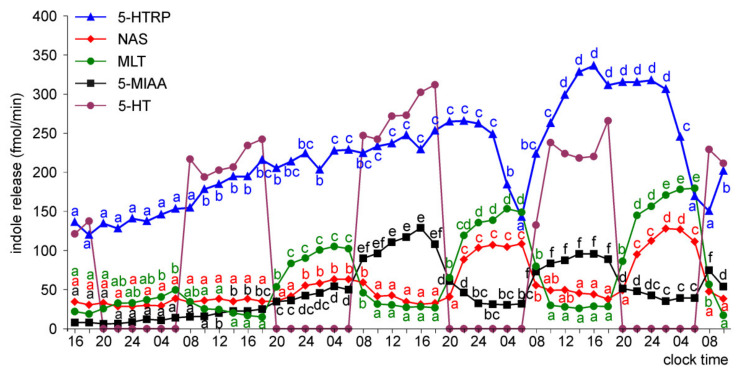
Release of 5-HTRP, NAS, MLT, and 5-MIAA (means, *n* = 3) from pineal organs of ED 20 embryos incubated under a 12L:12D cycle and treated with NE during photophase. The same letters indicate means, which are not significantly different between time-points.

**Figure 13:**
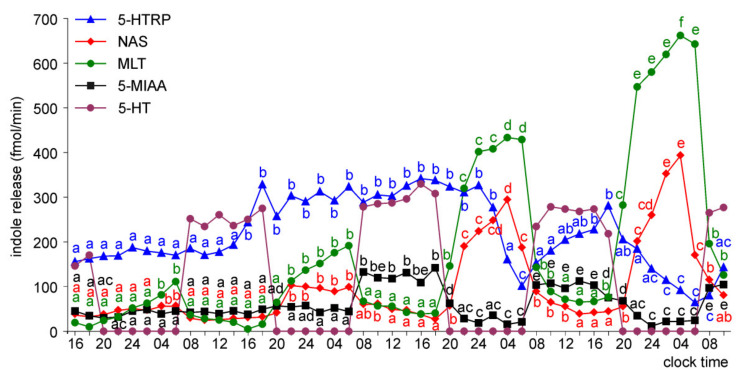
Release of 5-HTRP, NAS, MLT, and 5-MIAA (means, *n* = 3) from pineal organs of ED 22 embryos incubated under a 12L:12D cycle and treated with NE during photophase. The same letters indicate means, which are not significantly different between time-points.

The release patterns of the investigated indoles from the pineal organs of ED 20 and ED 22 embryos were generally similar to those described above, except for the presence of significant increases in NAS and MLT release during the first day of culture (Figure 12 and Figure 13). Moreover, in contrast to ED 18 embryos, the level of 5-MIAA was much above the detection and quantification limits in ED 20 and ED 22 embryos throughout the culture.

**Figure 14:**
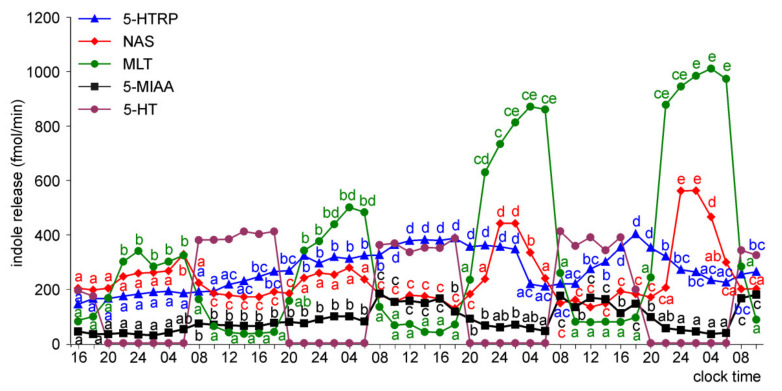
Release of 5-HTRP, NAS, MLT, and 5-MIAA (means, *n* = 3) from pineal organs of ED 24 embryos incubated under a 12L:12D cycle and treated with NE during photophase. The same letters indicate means, which are not significantly different between time-points.

The prominent daily changes in NAS and MLT release were noted starting from the first day of culture of the pineal organs of ED 24 embryos (Figure 14). The NAS and MLT release peaks were higher on days 3 and 4 of incubation than during the first two days. Moreover, the nocturnal peaks of NAS were narrower than the MLT peaks in the second half of the experiment. As in younger embryos, daily fluctuations in 5-HTRP and 5-MIAA release were restricted to days 3 and 4 of the culture. NE treatment induced a prominent increase in 5-HT release. 

**Figure 15:**
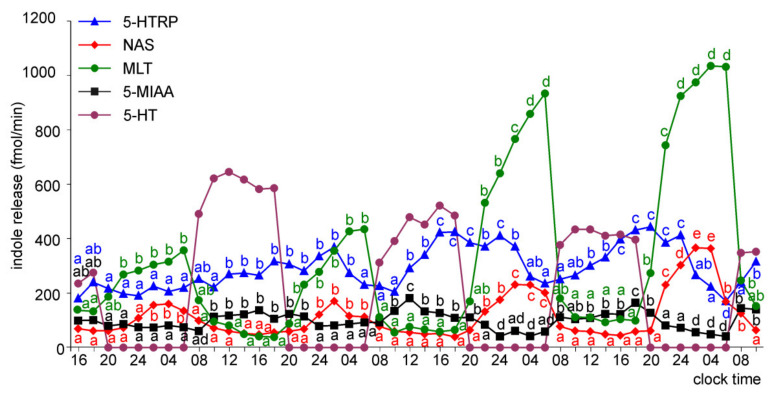
Release of 5-HTRP, NAS, MLT, and 5-MIAA (means, *n* = 3) from pineal organs of ED 26 embryos incubated under a 12L:12D cycle and treated with NE during photophase. The same letters indicate means, which are not significantly different between time-points.

**Figure 16:**
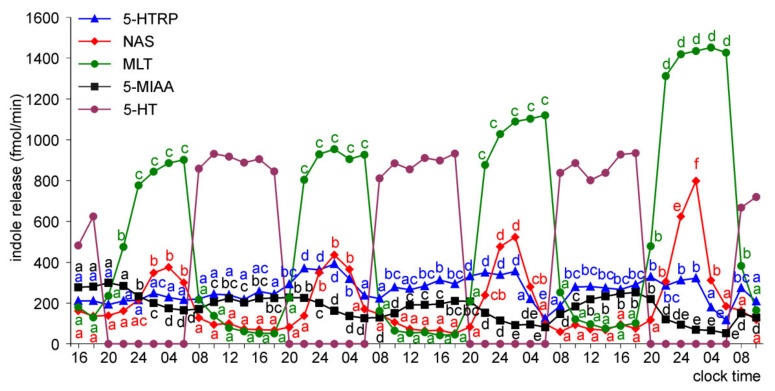
Release of 5-HTRP, NAS, MLT, and 5-MIAA (means, *n* = 3) from pineal organs of ED 28 embryos incubated under a 12L:12D cycle and treated with NE during photophase. The same letters indicate means, which are not significantly different between time-points.

The superfusion cultures of the pineal organs of ED26 and ED28 embryos were characterized by significant daily changes in the release of 5-HT, NAS, MLT, and 5-MIAA throughout the experiment (Figure 15 and Figure 16). Daily fluctuations in the release of 5-HTRP occurred from the second day of culture. The amplitudes of daily fluctuations of 5-HTRP and 5-MIAA release were much lower than those of 5-HT, NAS, and MLT release. The nocturnal NAS and MLT release significantly increased during the course of culture. Throughout the experiment, NAS peaks were narrower than the corresponding MLT peaks. In contrast to NAS and MLT, the release of 5-MIAA did not increase on consecutive days of culture. Like in younger embryos, the release of 5-HT was highly regular and showed a high amplitude of changes from a level below 10 fmol/min to 900 fmol/min. 

The release of 5-hydroxyindoles and 5-methoxyindoles from the pineal organs increased with embryo age, but there were some exceptions, such as higher levels of NAS release in the pineal organs of ED 24 embryos than in those of ED 26 embryos (Supplementary Materials, Figures S5–S9). Similar to the explants unexposed to NE, there were important differences in the ratio between indoles released into the culture medium depending on embryo age and the incubation period.

#### 2.1.2. Experiment II

**Figure 17:**
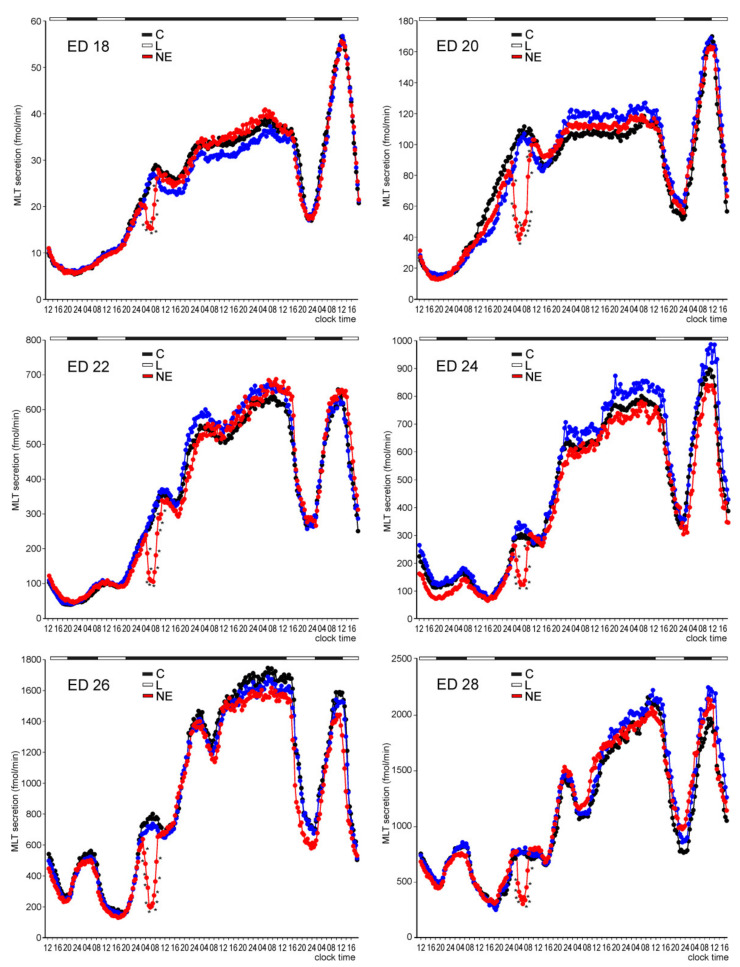
Secretion of MLT (means, *n* = 4) from pineal organs collected from the embryos at ED 18–ED 28 and incubated for 2 days under a 12L:12D cycle with photophase from 07.00 to 19.00, then for 2 days in continuous darkness, and next again under a 12L:12D cycle with photophase from 12.00 to 24.00. During the second day of culture between 02.00 and 06.00, the explants were exposed to the light with an intensity of 100lx (L), incubated in the medium containing at a NE concentration of 10 μM (NE) or left untreated (C). Asterisks indicate significant differences between the pineal organs treated with NE and those exposed and unexposed to light.

As in experiment I, the nocturnal peak of MLT secretion was absent on the first day of culture in the pineal organs of ED 18–ED 22 embryos (Figure 17). This peak occurred during the second day of culture, and at this time, the pineal organs from the experimental groups were exposed to light or treated with NE between 02.00 and 06.00. Light exposure was ineffective, whereas treatment with NE induced a rapid and significant decrease in MLT secretion. The culture in continuous darkness during the next two days resulted in a stepwise increase in MLT secretion, which did not differ between the control and experimental pineal organs. Incubation under 12L:12D cycle with photophase between 12:00 and 24:00 in the last phase of the experiment induced prominent daily changes in MLT secretion. 

The nocturnal peaks of MLT secretion were observed both on the first and second days of culture in the pineal organs of ED 24–ED 28 embryos (Figure 17). Similar to the pineal organs of younger embryos, the 4-h-long exposure to light during the second night did not change MLT secretion, while treatment with NE had a strong inhibitory effect on this process. The peak of MLT secretion also occurred during the natural night on the third day of culture (continuous darkness) in the pineal organs of ED 26 and ED 28 embryos. The course of MLT secretion in the last part of the experiment was similar to that observed in ED 18–ED 22 embryos.

### 2.2. In Vivo Study

**Figure 18:**
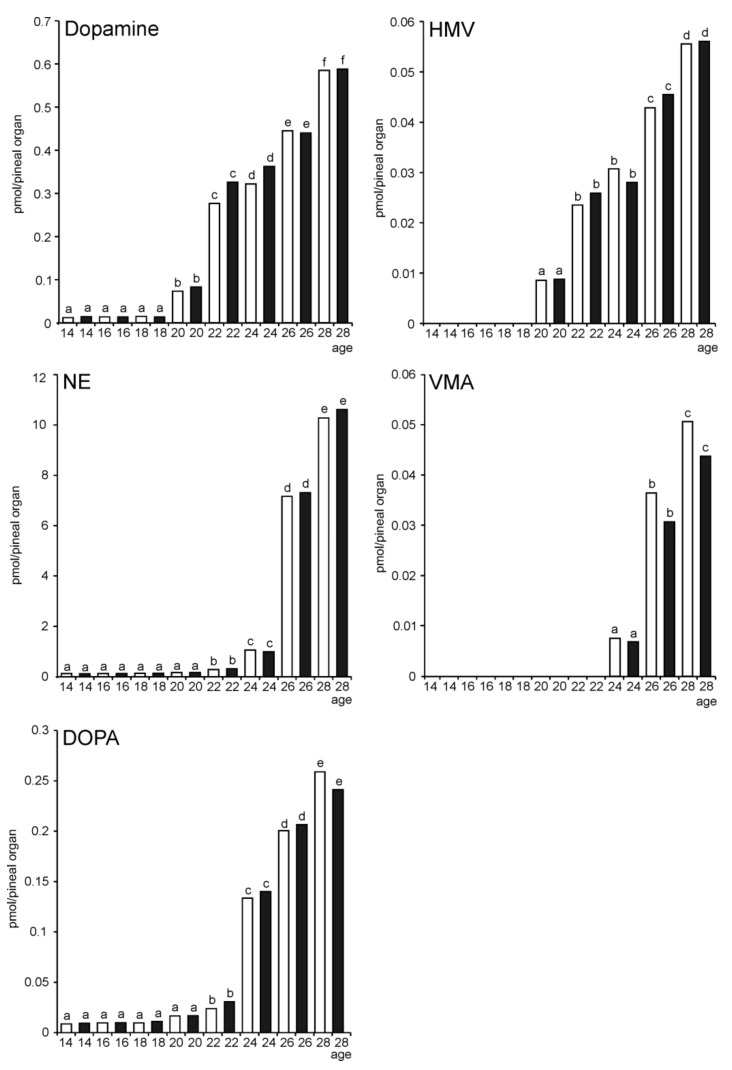
Content (means and SEM, *n* = 5) of norepinephrine (NE), vanillylmandelic acid (VMA), dopamine, homovanillic acid (HVA), and DOPA in the pineal organs of geese embryos at 14.00 (white columns) and 02.00 (black columns).

The NE content was very low in the embryonic pineal organs until ED 24, and a large increase in the level of this catecholamine was noted in ED 26 and ED 28 embryos (Figure 18). Vanillylmandelic acid (VMA), the main metabolite of NE, was undetectable in ED 14–ED 22 embryos, but its level prominently increased in ED 26 and ED 28 embryos. The dopamine levels and its metabolite, homovanillic acid (HVA), started to increase earlier in the embryonic development of goose pineal organs than NE and VMA. There were no daily fluctuations in catecholamines and their metabolites contents in the investigated pineal organs (Supplementary Materials, Figures S10–S12). 

## 3. Discussion

The study of the goose embryonic pineal organs in superfusion culture provided several new data on the ontogeny of pineal secretory activity in birds. Moreover, it revealed that isolation of these pineals from the in vivo environment promotes maturation of the MLT synthesis pathway and its daily rhythmicity.

The results obtained showed that the pineal organs of goose embryos at 18 days of age, ten days before hatching, secrete measurable amounts of MLT when placed in superfusion culture. Our previous study where MLT content was assayed in the pineal organs in vivo showed that MLT synthesis starts in the domestic goose between ED 17 and ED 18 [11]. However, it should be noted that both the secretion of MLT in vitro and the pineal MLT content in vivo, despite some increase with age, were very low between ED 18 and ED 22, and dramatically increased in the pineal organs of ED 24 embryos. Therefore, the last 5–6 days of embryonic life (ED 24–ED 28) should be considered as a period when the goose pineal organ may play a role as an endocrine gland that secretes MLT.

The comparative analysis of data on the ontogeny of MLT secretion in the goose and the chicken should consider the differences in the length of embryonic development (goose: 29 days, chicken: 21 days) [34]. The chicken pineal organs of ED 10 embryos released only traces, hardly detectable amounts of MLT into the culture medium during a 24-h-long static incubation [5]. The release of MLT increased gradually with embryo age up to ED 17. A prominent increase in MLT secretion occurred just before hatching between ED 17 and ED 19. In superfusion culture, MLT secretion was demonstrated in the chicken pineal organs of ED 13 embryos; however, it was approximately 10-fold lower than that in the pineal organs of ED 18 embryos [2,33]. It seems that the period with a considerably high MLT secretion during embryonic development is slightly longer in the goose than in the chicken.

For the first time in ontogeny, the daily changes in MLT secretion in superfusion culture under a 12L:12D cycle were observed in the pineal organs of ED 24 embryos. It should be noted that the significant daily fluctuations of the pineal MLT content in vivo were detected earlier, in ED 20 and ED 22 goose embryos, where the hormone levels were higher at the end of the scotophase than during the photophase [11]. For chicken, the day-night differences in MLT release were found in the pineal cell cultures obtained from 13- and 14-day-old embryos under a 12L:12D cycle [30]. However, the daily rhythm of MLT secretion was not found in static cultures (in a 12L:12D cycle) of the pineal organs of ED 14 and ED 15 chicken embryos [4]. This rhythm was first noted in ED 16 embryos, but not in all explants. Daily changes in the pineal content of MLT were reported in 19-day-old chick embryos maintained under 12L:12D and 16L:8D, but not under 8L:16D [7]. Such changes in AA-NAT mRNA content were described in the chicken pineal organs of 16- and 19-day-old embryos [8,35], while in AA-NAT activity in 17-, 18-, and 19-day-old embryos [36,37]. Plasma MLT concentrations were measurable in ED 15 [38] and showed daily fluctuations, depending on the photophase period, in ED 19 chicken embryos [7,39].

Our results showed that the secretory activity of the embryonic pineal organs changed significantly during the course of superfusion culture performed under the 12 L:12D cycle. On the second day of culture, prominent nocturnal peaks of MLT secretion occurred in all studied pineal organs, including those of ED 18–ED 22 embryos. The levels of MLT secretion at daytime nadirs and nocturnal peaks prominently increased on consecutive days. The elevation of MLT secretion in the course of culture was larger in the pineal organs of younger embryos than older embryos. These changes cannot be explained as a simple continuation of the developmental processes in superfusion culture because they occurred much faster than in vivo. For example, the nocturnal secretion of MLT and the amplitude of daily changes in the pineal organs of ED 22 embryos on the third day of culture were much higher than in those of ED 24 embryos on the first day of culture. Therefore, it could be concluded that the isolation of the embryonic pineal organs from their niche induced the development of the MLT secretory machinery and its daily rhythmicity. To the best of our knowledge, this phenomenon has not been previously reported. It should be noted that culture was performed in a medium without the addition of serum or proteins.

To characterize changes in pineal metabolism occurring during embryonic development and in the course of superfusion culture, we measured the concentrations of 5-HTRP, 5-HT, NAS, MLT, and 5-MIAA in the selected medium samples by HPLC with fluorescence detection. The levels of other pineal 5-hydroxyindoles and 5-methoxyindoles could not be estimated because their peaks were unresolved from the medium components or below the detection limit. The present study is the first to describe the release of 5-HTRP, 5-HT, NAS, and 5-MIAA from the avian pineal organ in superfusion culture. It should be emphasized that the permeability of indoles across the cell membrane differs greatly among compounds, and the proportion of indoles in the culture medium does not reflect that within pinealocytes. The analysis of indole release during the first day of culture clearly shows that the development of the 5-HT synthesis pathway is the first step in the ontogenesis of indole metabolism. The pineal organs of ED 18 embryos released 5-HTRP in the daily rhythm, and the release of this compound was approximately 10-folds higher than that of MLT. The rhythmic release of 5-HTRP also occurred in older embryos; however, there was a shift in the ratio between 5-HTRP and MLT. The predominance of 5-HTRP over MLT decreased with the age of embryos, and finally, the release of MLT in the pineal organs of ED 26 and ED 26 was much higher than that of 5-HTRP. Furthermore, the changes in the ratio between NAS and MLT released into the medium suggest that the synthesis of NAS develops faster than the capability of this indole methylation. The release of NAS was higher than that of MLT in the pineal organs of ED 18 embryos, but lower than that of MLT in the organs of ED 22–ED 28 embryos. The results obtained in superfusion culture agree with those from measurements of the indole content in the goose pineal organs [11] and support the idea that MLT synthesis develops during ontogeny in three stages, which are turned on one after another: (1) 5-HT synthesis, (2) acetylation of 5-HT into NAS, and (3) methylation of NAS.

The changes in indole release occurring in the course of culture were generally similar to those related to the age of the embryos. They involved (i) the establishment of the rhythmic changes in the release of NAS, MLT, and 5-MIAA, (ii) an increase in the ratio between MLT and 5-HTRP release, and (iii) the changes in the ratio between the release of MLT and NAS. The daily rhythm of 5-HTRP release was maintained throughout the culture period in all explants, while the rhythmic changes of NAS, MLT, and 5-MIAA release developed with a delay in the pineal organs of ED 18–ED 22 embryos. The release of 5-HTRP decreased in the course of culture in the pineal organs of ED 18–ED 22 embryos or remained the same on consecutive days in the pineal organs of ED 24–ED 28, while the secretion of MLT increased more or less prominently. MLT to NAS release ratio increased during culture in the pineal organ of ED 18 and ED 20 embryos.

The superfusion culture allowed us to study the factors regulating the secretory activity of the goose pineal organ during embryonic development. The comparison of MLT secretion under normal 12L:12D cycle and reversed 12D:12L cycle, and in continuous darkness, shows that the goose embryonic pineal organs are directly photosensitive, and the daily alternations of light-dark phases drive rhythmic MLT secretion. The secretory activity was entrained to the reversed light-dark cycle on the second day of culture in the pineal organs of all age groups of embryos. According to our previous study, this also takes 2 days in the pineal organs of 14-week-old turkeys [21] and 1-day-old ducks, but 3 days in 9-month-old ducks [40] and 12-week-old geese [12]. In chicken embryonic pineal organs, the rhythm is entrained to the reversed cycle on the second day of culture [33]. Continuous darkness caused a long-term increase in MLT secretion from the goose embryonic pineal organs; however, restoration of the light-dark dark cycle induced rhythmic secretion of the pineal hormone.

Special attention should be paid to our results, which showed that a 4-h-long exposition to light during scotophase did not decrease MLT secretion nor changed the course of pineal secretion in the following incubation period in continuous darkness. These data demonstrate that the direct action of light on the pineal organs of goose embryos is limited in some way, possibly to specific phases of the daily cycle. A study performed on the pineal organs of 12-week-old geese in superfusion culture showed that light alone was not sufficient for the precise entrainment of MLT rhythm to the light-dark cycle [16]. Similar data were obtained in studies on the pineal organs of adult domestic ducks [40]. We suppose that light reception occurs in the pineal organs of *Anseriformes* birds via melanopsin, which is functionally connected with the regulation of gene expression. To date, our attempts to detect pinopsin in the goose and duck pineal organs have been unsuccessful (unpublished data). Several experiments performed in chickens demonstrated the importance of light in regulating pineal activity during embryonic development [6,7,38,39,41]. However, given our data, special attention should be paid to the results showing that exposure to 2 h light pulses did not decrease the nocturnal melatonin content in the pineal organ of ED 19 chicken embryos [10]. Three hours of light effectively reduced the pineal melatonin content when applied at ZT 21–24 and during the subjective light phase, but not at ZT 18–21 (in constant darkness). In hatchlings, light pulses decreased the MLT content independently of daily time.

Continuous darkness incubation was performed to determine whether an endogenous oscillator was active in the embryonic pineal organs. Under these conditions, the MLT secretion peaks occurred exclusively on the first day of culture in the pineal organs of ED 24–ED 28 embryos, demonstrating that the endogenous oscillator works in these explants, but it is unstable in vitro and quickly loses its ability to affect MLT secretion. The data obtained during incubation of the pineal organs of younger embryos indicate two possibilities: (i) absence of endogenous oscillator activity or (ii) no functional connection between the oscillator and synthesis of NAS and MLT. Similar to the present data, studies on the pineal organs of 12-week-old geese showed that the endogenous oscillator lost its activity after 1 or 2 days of culture [16]. Additionally, the activity of the endogenous oscillator in embryonic chicken pineal organs or isolated cells was not sustained for more than two days [8,30].

Our study showed that embryonic goose pinealocytes are sensitive to NE, which induces a rapid and large decrease in MLT secretion. The treatment of explants with NE during the photophases of 12L:12D cycle resulted in a high-amplitude, well-entrained rhythm of MLT secretion. The effects of NE were observed even in the pineal organs of ED 18 embryos. Our results are the first to demonstrate the strong inhibitory action of NE on MLT secretion during photophase in avian embryonic pinealocytes. Lamošová et al. [4] found that NE decreased MLT secretion from chicken pineal cells of 19-day-old embryos in culture by ca. 50% during the dark phase of the light-dark cycle; however, NE was completely ineffective during the photophase. In post-hatching chickens, NE inhibits MLT synthesis by decreasing cAMP levels [42,43]; therefore, it is worth noting that forskolin, 8-bromocyclic AMP, PACAP, and VIP increased MLT secretion in embryonic chicken pinealocytes [4,30,31,33,44,45], demonstrating that cAMP is involved in the control of the activity of these cells.

The analysis of data on the indole release obtained using HPLC revealed that treatment with NE induced large changes in pineal metabolism. NE evoked a large and rapid increase in the release of 5-HT in the pineal organs of all studied embryos. Such an effect has not been described in the avian pineal organs. Initially, treatment with NE abolished the rhythmic changes in 5-HTRP and 5-MIAA release; however, these rhythms were restored in the second part of the experiment. This effect was probably caused by a temporary disturbance of homeostasis in indole metabolism or the direct effect of NE on the activity of tryptophan hydroxylase.

The sensitivity of pinealocytes to NE led to the question of the presence of this neurotransmitter in embryonic pineal organs. To answer this question, the contents of catecholamines and their metabolites were measured in the pineal organs of embryos by HPLC with electrochemical detection. The NE level increased significantly at the end of embryonic development; however, no daily changes were observed in the NE and its metabolite, VMA. Such variations were reported in *Anseriformes* birds at several weeks of age [16,40]. Our results suggest that even NE is not involved in regulating goose pineal activity in embryos, such regulation may start shortly after hatching. It should be noted that NE is a crucial factor controlling MLT secretion in the pineal organ of geese [16] and ducks [40] at the age of several weeks. In literature, it is often mentioned that the post-hatching structural and functional modifications of the avian pineal organ involve reducing the sensory structures and developing the sympathetic input [46]. The present study clearly shows that adrenergic regulation of pineal function develops in some birds at the end of embryonic life.

## 4. Materials and Methods

### 4.1. Embryos

Domestic goose (*Anser anser f. domestica*) eggs were obtained from a local reproductive farm and incubated in an Ova-Easy 580 Advance Series II incubator (Brinsea, Titusville, FL, USA) as previously described [11]. The incubation was performed under 12 h of light and 12 h of darkness cycle with a photoperiod from 07:00 to 19:00. The intensity of light provided by light-emitting diodes (5000 K) on the eggshell was approximately 20 lx. All experimental procedures on embryos were performed following Polish and EU laws (project No. 2018/31/N/NZ4/01248, 15 January 2015).

### 4.2. In Vitro Study

#### 4.2.1. Culture Medium

Medium 199 containing Earle’s salt and HEPES (Sigma-Aldrich, St. Louis, MO, USA) was prepared from a powder according to the manufacturer’s instructions (pH adjusted to 7.2 with NaOH) and sterilized by filtration. Before use, the sterile solution of ascorbic acid (Sigma-Aldrich, St. Louis, MO, USA) in Medium 199 and Antibiotic-Antimycotic Solution (Sigma-Aldrich, St. Louis, MO, USA) were added to give a final concentration 300 mg/L of ascorbic acid, 100 IU/mL of penicillin, 100 μg/mL of streptomycin, and 0.25 μg/mL of amphotericin B. A 1 mM solution (freshly prepared) of norepinephrine bitartrate (Sigma-Aldrich, St. Louis, MO, USA) was sterilized by filtration and diluted in the culture medium to 10 μM.

#### 4.2.2. Superfusion Culture Technique

The pineal organs were isolated directly after decapitation between 09:00 and 11:00, wrapped in nylon mesh, and placed individually in culture chambers connected to a multichannel peristaltic pump (Cole Parmer, Vernon Hills, IL, USA) and a manual fraction collector by their upper pools. The medium, continuously equilibrated with a gas mixture of 95% 02 and 5% CO2, flowed through a lower pool to the culture chambers at 0.05 mL/min. Incubation was performed at 38.5 °C in a water bath (Julabo, Seelbach, Germany). The chambers were illuminated with fluorescent lamps emitting white light with 100 lx intensity on the chamber surface during the photophase. During the scotophase, lightproof polyethylene covers were placed on the culture chambers. Fractions of medium flowing out of the superfusion culture system were collected every 30 min and frozen until assays were performed. The superfusion system enabled the simultaneous incubation of 24 explants in a separate culture chamber.

#### 4.2.3. Experiment I

The pineal organs of embryos at the age of 18, 20, 22, 24, 26, and 28 days were incubated for 4 days under a 12L:12D cycle with photophase from 07.00 to 19.00, a 12L:12D cycle, but in the culture medium containing NE 10 μM during the photophase (from 07.00 to 19.00), a reversed dark-light cycle with photophase from 19:00 to 07:00, or continuous darkness. The experiment consisted of four identical replicates with respect to the age of the embryos and the protocol (96 pineal organs in total, 4 per age/treatment group).

#### 4.2.4. Experiment II

The pineal organs of embryos at the ages of 18, 20, 22, 24, 26, and 28 days were incubated for 2 days under a 12L:12D cycle with photophase from 07.00 to 19.00, then for 2 days in a continuous darkness, and then again under a 12L:12D cycle, but with photophase from 12.00 to 24.00. During the second day of culture between 02.00 and 06.00, the pineal organs were exposed to light with 100 lx intensity on the chamber surface, incubated in the medium containing at NE a concentration of 10 μM or left untreated (control). The experiment was divided into three parts; each part involved explants of all age groups and all kinds of treatment in each age group (72 pineal organs in total, 4 per age/treatment group).

### 4.3. In Vivo Study

The pineal organs were dissected immediately after the decapitation at 14.00 and 02.00 from embryos at the age of 14, 16, 18, 20, and 22 days, and at 8.00, 10.00, 12.00, 14.00, 16.00, 18.00, 20.00, 22.00, 24.00, 02.00, 04.00, and 06.00 from embryos at the age of 24, 26, and 28 days. Five pineal organs per time point were collected from each age group. As previously described, decapitation and dissection of the pineal organs during the scotophase were performed with minimal exposure to light [11]. The pineal organs were immediately frozen at −80 °C and stored until the assay of catecholamines and their metabolites.

### 4.4. Analytical Procedures

#### 4.4.1. Chemicals

The anti-melatonin antibody Prospect 6C was kindly provided by Dr. Andrew Foldes (Division of Animal Production, CSIRO, Blacktown, Australia), 3H-melatonin was purchased from PerkinElmer (Waltham, MA, USA), gelatin from Merck (Billerica, MA, USA), and toluene from POCH, Gliwice Poland). Chemicals of the highest purity were purchased from J. T. Baker Chemicals (Center Valley, PA, USA) and used to prepare the mobile phases: sodium acetate, sodium dihydrogen phosphate, disodium EDTA, 1-octanesulfonic acid sodium salt, citric acid, acetic acid, and phosphoric acid. Methanol and acetonitrile, both of gradient-grade HPLC purities, were purchased from Merck Millipore (Billerica, MA, USA). 5-HTOL was provided by Santa Cruz Biotechnology (Dallas, TX, USA). All other reagents were purchased from Sigma-Aldrich (St. Louis, MO, USA). Ultrapure water (18.2 MΩ, TOC ≤ 3 ppb) was freshly prepared using a Milli-Q^®^ IQ 7003/05 purification system (Merck Millipore, Billerica, MA, USA) and was used in all procedures.

#### 4.4.2. Melatonin Radioimmunoassay

The MLT concentration in the medium samples was measured by direct RIA with Prospect 6C antiserum and 3H-melatonin (3.2 TBq/mmol) as previously described and characterized in detail [21]. The volume of the medium used in the assay varied from 40 to 400 μL because of the age-dependent differences in MLT secretion. The sensitivity of the assay was better than 10 pg/tube, and the intra- and inter-assay coefficients of variation were below 10%.

#### 4.4.3. Assay of 5-Hydroxyindoles and 5-Methoxyindoles in Culture Medium Samples

The medium samples were thawed at temperatures below 8 °C, vortexed, and 450 μL of the sample was transferred to an Eppendorf tube, to which 50 μL of perchloric acid was added. The mixture was vortexed, incubated in an ice bath for 20 min, and centrifuged at 30,000 × g (4 °C) for 15 min (Allegra 64R, Beckman Coulter, Indianapolis, IN, USA). Next, 400 μL of liquid was transferred to an autosampler vial.

The indole content in the culture medium was measured using an HPLC method based on gradient-grade separation and fluorescence detection with programmed sensitivity changes. The assay was performed on a Vanquish Duo U/HPLC system equipped with two gradient pumps, a cooled dual-split autosampler, column compartment, and two fluorescence detectors (Thermo Fisher Scientific, Waltham, MA, USA). The autosampler was maintained at 6 °C. The injection volume was 100 µL. Hypersil Gold aQ columns with 3 µm particle size and dimensions of 150 × 4.6 mm (Thermo Fisher Scientific, Waltham, MA, USA), as well as the mobile phase prepared through on-line mixing of methanol and a water solution of 5 mM sodium acetate and 0.01 mM disodium EDTA (pH 4.5 by adding acetic acid) were used to separate the analyzed compounds. The flow rate of the mobile phase was 1 mL/min. The initial methanol concentration was 2% (*v*/*v*). Between the 7th and 20th min of separation, the methanol content was increased linearly to 30% (*v*/*v*), maintained at a constant level for 10 min, and then decreased to the initial level. The detection was performed at an excitation wavelength of 280 nm and an emission wavelength of 345 nm at a constant temperature of 38 °C. The sensitivity of the detector was changed at the 5.8th min of the separation from level 1 to 8, at 12th min of separation again to level 1 and at 15.7th min of separation to level 8. Chromatograms were analyzed using Chromeleon 7.2.10 software (Thermo Fisher Scientific, Waltham, MA, USA). The limits of quantification (S/N ratio of 10:1) for all measured indoles was below 4 pg per injection. The intra-day precision was below 2%, and the inter-day precision was below 3%.

#### 4.4.4. Assay of Catecholamines and Their Metabolites

The pineal organs were sonicated in 80 μL of ice-cold 0.1 M perchloric acid using a Vibra-Cell VC 70 ultrasonic processor with a 2-mm probe (Sonics & Materials Inc., Newtown, CT, USA), and the homogenates were incubated for 15 min in an ice bath. After centrifugation at 30,000× *g* (4 °C) for 15 min (Allegra 64R, Coulter Beckman, Indianapolis, IN, USA), the supernatants were used to measure the catecholamines and their metabolites, which was performed by HPLC with electrochemical detection as described previously [14].

### 4.5. Statistical Analysis

The data from experiment I were analyzed using repeated-measures analysis of variance with the treatment as the main effect and the sampling time as a repeated measure. The least significant difference test (LSD) was used as a post-hoc test. The data from experiment II were subjected to a one-way analysis of variance with Duncan’s test. The data from the in vivo studies were analyzed by two-way analysis of variance with age and time as factors. Duncan’s test was applied as a post-hoc procedure. A value of *p* < 0.05, was considered significant in all statistical procedures. The analyses were performed using Dell Statistica 13 (Version 13.1 PL, Dell Inc., Tulsa, OK, USA).

## 5. Conclusions

Due to the specific physiological features of the goose pineal organ and the use of novel analytical methods and experimental protocols, this study provided several new data on the embryonic development of the avian pineal organs. For the first time, it has been demonstrated that the rhythmic release of 5-HTRP from the pineal organs in vitro occurs several days before the development of daily rhythm in the release of NAS, MLT, and 5-MIAA. The analysis of age-dependent changes in the indole release supports our previous hypothesis that MLT synthesis develops during ontogeny in three stages, which are turned on one after another: (1) 5-HT synthesis, (2) acetylation of 5-HT into NAS, and (3) methylation of NAS. Moreover, the results showed that isolation of the embryonic pineal organs from their niche induced the development of the MLT secretory machinery and its daily rhythmicity. This finding is important when analyzing the results obtained in long-term cultures of avian embryonic pineal cells, such as monolayer cultures. The modifications of indole metabolism occurring during the course of culture were similar to age-dependent changes. Regarding the regulatory mechanisms, this study demonstrated the strong inhibitory action of NE on MLT secretion by embryonic pineal cells, both during the photophase and the scotophase. The treatment of explants with NE during consecutive photophases resulted in a high-amplitude, well-entrained rhythm of MLT secretion. This study also demonstrated for the first time that NE induces a large increase in the release of 5-HT from the avian pineal organ. The increase in the content of this catecholamine on the last days of embryonic life suggests that adrenergic regulation of pineal activity may develop before or shortly after hatching. The study also showed a lack of effect of 4-h-long light exposure of the pineal organs during scotophase on MLT secretion. However, it should be stressed that MLT secretion was controlled directly by the light-dark cycle in all studied age groups of explants. In addition, the endogenous oscillator was active in the pineal organs of embryos at the age of 24 days and older but could not generate a rhythm after one cycle.

## Data Availability

All data used to support the findings of this study are included within the article. Raw data can be available on request.

## Supplementary Materials

The following are available online, Figure S1. Comparison of 5-HTRP release from the pineal organs of ED 18–ED 28 embryos incubated under a 12L:12D cycle. Figure S2. Comparison of NAS release from the pineal organs of ED 18–ED 28 embryos incubated under a 12L:12D cycle. Figure S3. Comparison of MLT release from the pineal organs of ED 18–ED 28 embryos incubated under a 12L:12D cycle. Figure S4. Comparison of 5-MIAA release from the pineal organs of ED 18–ED 28 embryos incubated under a 12L:12D cycle. Figure S5. Comparison of 5-HTRP release from the pineal organs of ED 18–ED 28 embryos incubated under a 12L:12D cycle and treated with NE during photophase. Figure S6. Comparison of NAS release from the pineal organs of ED 18–ED 28 embryos incubated under a 12L:12D cycle and treated with NE during photophase. Figure S7. Comparison of MLT release from the pineal organs of ED 18–ED 28 embryos incubated under a 12L:12D cycle and treated with NE during photophase. Figure S8. Comparison of 5-MIAA release from the pineal organs of ED 18–ED 28 embryos incubated under a 12L:12D cycle and treated with NE during photophase. Figure S9. Comparison of 5-HT release from the pineal organs of ED 18–ED 28 embryos incubated under a 12L:12D cycle and treated with NE during photophase. Figure S10. Content of norepinephrine (NE), vanillylmandelic acid (VMA), dopamine (DA), homovanillic acid (HVA), and DOPA in the pineal organs of ED 24 embryos collected every 2-h. Figure S11. Content of norepinephrine (NE), vanillylmandelic acid (VMA), dopamine (DA), homovanillic acid (HVA), and DOPA in the pineal organs of ED 26 embryos collected every 2-h. Figure S12. Content of norepinephrine (NE), vanillylmandelic acid (VMA), dopamine (DA), homovanillic acid (HVA), and DOPA in the pineal organs of ED 28 embryos collected every 2-h.

## Sample Availability

Samples of the compounds are not available from the authors.

## References

[1] Drozdova A., Okuliarova M., Zeman M. (2019). The effect of different wavelengths of light during incubation on the development of rhythmic pineal melatonin biosynthesis in chick embryos. Animal.

[2] Csernus V.J., Nagy A.D., Faluhelyi N. (2007). Development of the rhythmic melatonin secretion in the embryonic chicken pineal gland. Gen. Comp. Endocrinol..

[3] Bai X., Cao J., Dong Y., Wang Z., Chen Y. (2019). Melatonin mediates monochromatic green light-induced satellite cell proliferation and muscle growth in chick embryo. PLoS ONE.

[4] Lamosová D., Zeman M., Macková M., Gwinner E. (1995). Development of rhythmic melatonin synthesis in cultured pineal glands and pineal cells isolated from chick embryo. Experientia.

[5] Möller W., Möller G. (1990). Structural and functional differentiation of the embryonic chick pineal organ in vivo and in vitro. A scanning electron-microscopic and radioimmunoassay study. Cell Tissue Res..

[6] Zeman M., Gwinner E., Somogyiová E. (1992). Development of melatonin rhythm in the pineal gland and eyes of chick embryo. Experientia.

[7] Zeman M., Gwinner E., Herichová I., Lamosová D., Kost’ál L. (1999). Perinatal development of circadian melatonin production in domestic chicks. J. Pineal Res..

[8] Herichová I., Zeman M., Macková M., Griac P. (2001). Rhythms of the pineal N-acetyltransferase mRNA and melatonin concentrations during embryonic and post-embryonic development in chicken. Neurosci. Lett..

[9] Zeman M., Pavlik P., Lamosová D., Herichová I., Gwinner E. (2004). Entrainment of rhythmic melatonin production by light and temperature in the chick embryo. Avian Poult. Biol. Rev..

[10] Herichová I., Monosíková J., Zeman M. (2008). Ontogeny of melatonin, Per2 and E4bp4 light responsiveness in the chicken embryonic pineal gland. Comp. Biochem. Physiol. Part A Mol. Integr. Physiol..

[11] Hanuszewska M., Prusik M., Lewczuk B. (2019). Embryonic Ontogeny of 5-Hydroxyindoles and 5-Methoxyindoles Synthesis Pathways in the Goose Pineal Organ. Int. J. Mol. Sci..

[12] Ziółkowska N., Lewczuk B., Prusik M. (2018). Diurnal and circadian variations in indole contents in the goose pineal gland. Chronobiol. Int..

[13] Adamska I., Lewczuk B., Markowska M., Majewski P.M. (2016). Daily profiles of melatonin synthesis-related indoles in the pineal glands of young chickens (*Gallus gallus domesticus* L.). J. Photochem. Photobiol. B Biol..

[14] Lewczuk B., Ziółkowska N., Prusik M., Przybylska-Gornowicz B. (2014). Diurnal profiles of melatonin synthesis-related indoles, catecholamines and their metabolites in the duck pineal organ. Int. J. Mol. Sci..

[15] Grady R.K., Caliguri A., Mefford I.N. (1984). Day/night differences in pineal indoles in the adult pigeon (*Columba livia*). Comp. Biochem. Physiol. C Comp. Pharmacol. Toxicol..

[16] Ziółkowska N., Lewczuk B. (2021). Norepinephrine Is a Major Regulator of Pineal Gland Secretory Activity in the Domestic Goose (*Anser anser*). Front. Physiol..

[17] Takahashi J.S., Hamm H., Menaker M. (1980). Circadian rhythms of melatonin release from individual superfused chicken pineal glands in vitro. Proc. Natl. Acad. Sci. USA.

[18] Robertson L.M., Takahashi J.S. (1988). Circadian clock in cell culture: II. In vitro photic entrainment of melatonin oscillation from dissociated chick pineal cells. J. Neurosci..

[19] Csernus V., Faluhelyi N., Nagy A.D. (2005). Features of the circadian clock in the avian pineal gland. Ann. N. Y. Acad. Sci..

[20] Csernus V., Becher P., Mess B. (1999). Wavelength dependency of light-induced changes in rhythmic melatonin secretion from chicken pineal gland in vitro. Neuro. Endocrinol. Lett..

[21] Prusik M., Lewczuk B. (2019). Roles of Direct Photoreception and the Internal Circadian Oscillator in the Regulation of Melatonin Secretion in the Pineal Organ of the Domestic Turkey: A Novel In Vitro Clock and Calendar Model. Int. J. Mol. Sci..

[22] Martyniuk K., Hanuszewska M., Lewczuk B. (2020). Metabolism of Melatonin Synthesis-Related Indoles in the Turkey Pineal Organ and Its Modification by Monochromatic Light. Int. J. Mol. Sci..

[23] Przybylska-Gornowicz B., Lewczuk B., Prusik M., Nowicki M. (2005). Post-hatching development of the turkey pineal organ: Histological and immunohistochemical studies. Neuro. Endocrinol. Lett..

[24] Petrusewicz-Kosińska M., Przybylska-Gornowicz B., Ziółkowska N., Martyniuk K., Lewczuk B. (2019). Developmental morphology of the turkey pineal organ. Immunocytochemical and ultrastructural studies. Micron..

[25] Prusik M., Lewczuk B., Nowicki M., Przybylska-Gornowicz B. (2006). Histology and ultrastructure of the pineal gland of the domestic goose. Histol. Histopathol..

[26] Prusik M., Lewczuk B. (2008). Structure of the avian pineal gland. Med. Weter..

[27] Fejér Z., Röhlich P., Szél A., Dávid C., Zádori A., Manzano M.J., Vígh B. (2001). Comparative ultrastructure and cytochemistry of the avian pineal organ. Microsc. Res. Tech..

[28] Boya J., Calvo J. (1980). Ultrastructural study of the post-hatching evolution of the pineal gland of the chicken (*Gallus gallus*). Cells Tissues Organs.

[29] Boya J., Calvo J. (1979). Evolution of the pineal gland in the adult chicken. Cells Tissues Organs.

[30] Akasaka K., Nasu T., Katayama T., Murakami N. (1995). Development of regulation of melatonin release in pineal cells in chick embryo. Brain Res..

[31] Macková M., Lamosová D., Zeman M. (1998). Regulation of rhythmic melatonin production in pineal cells of chick embryo by cyclic AMP. Cell. Mol. Life Sci..

[32] Faluhelyi N., Csernus V. (2007). The effects of environmental illumination on the in vitro melatonin secretion from the embryonic and adult chicken pineal gland. Gen. Comp. Endocrinol..

[33] Faluhelyi N., Reglodi D., Csernus V. (2005). Development of the circadian melatonin rhythm and its responsiveness to PACAP in the embryonic chicken pineal gland. Ann. N. Y. Acad. Sci..

[34] Li S., Bai S., Qin X., Zhang J., Irwin D.M., Zhang S., Wang Z. (2019). Comparison of whole embryonic development in the duck (*Anas platyrhynchos*) and goose (*Anser cygnoides*) with the chicken (*Gallus gallus*). Poult. Sci..

[35] Kommedal S., Csernus V., Nagy A.D. (2013). The embryonic pineal gland of the chicken as a model for experimental jet lag. Gen. Comp. Endocrinol..

[36] Binkley S., Geller E.B. (1975). Pineal Enzymes in Chickens: Development of Daily Rhythmicity. Gen. Comp. Endocrinol..

[37] Zeman M., Illnerová H. (1990). Ontogeny of N-acetyltransferase activity rhythm in pineal gland of chick embryo. Comp. Biochem. Physiol. A Comp. Physiol..

[38] Wang T., Wang Z., Cao J., Dong Y., Chen Y. (2014). Monochromatic light affects the development of chick embryo liver via an anti-oxidation pathway involving melatonin and the melatonin receptor Mel1c. Can. J. Anim. Sci..

[39] Archer G.S., Mench J.A. (2014). The effects of the duration and onset of light stimulation during incubation on the behavior, plasma melatonin levels, and productivity of broiler chickens. J. Anim. Sci..

[40] Prusik M., Lewczuk B., Ziółkowska N., Przybylska-Gornowicz B. (2015). Regulation of melatonin secretion in the pineal organ of the domestic duck-an in vitro study. Pol. J. Vet. Sci..

[41] Faluhelyi N., Matkovits A., Párniczky A., Csernus V. (2009). The in vitro and in ovo effects of environmental illumination and temperature on the melatonin secretion from the embryonic chicken pineal gland. Ann. N. Y. Acad. Sci..

[42] Voisin P., Van Camp G., Collin J.P. (1990). N-acetylation of serotonin is correlated with alpha 2- but not with beta-adrenergic regulation of cyclic AMP levels in cultured chick pineal cells. J. Neurochem..

[43] Zawilska J.B., Berezińska M., Stasikowska O., Lorenc A., Skene D.J., Nowak J.Z. (2005). Posthatching developmental changes in noradrenaline content in the chicken pineal gland. J. Pineal Res..

[44] Faluhelyi N., Reglodi D., Lengvári I., Csernus V. (2004). Development of the circadian melatonin rhythm and the effect of PACAP on melatonin release in the embryonic chicken pineal gland. An in vitro study. Regul. Pept..

[45] Faluhelyi N., Reglodi D., Csernus V. (2006). The effects of PACAP and VIP on the in vitro melatonin secretion from the embryonic chicken pineal gland. Ann. N. Y. Acad. Sci..

[46] Sato T. (2001). Sensory and endocrine characteristics of the avian pineal organ. Microsc. Res. Tech..

