# Supplementary material for: Embryonic Development of Avian Pineal Secretory Activity—A Lesson from the Goose Pineal Organs in Superfusion Culture
Source: Molecules. 2021 Oct 20;26(21):6329. doi: 10.3390/molecules26216329 (PMC8588208; doi:10.3390/molecules26216329)
Supplement: Supplementary file 1 [file molecules-26-06329-s001.zip › molecules-1422240-supplementary.pdf]

**Supplementary Materials**
**Embryonic Development of Avian Pineal Secretory Activity—A Lesson from the Goose Pineal Organs in Superfusion Culture**

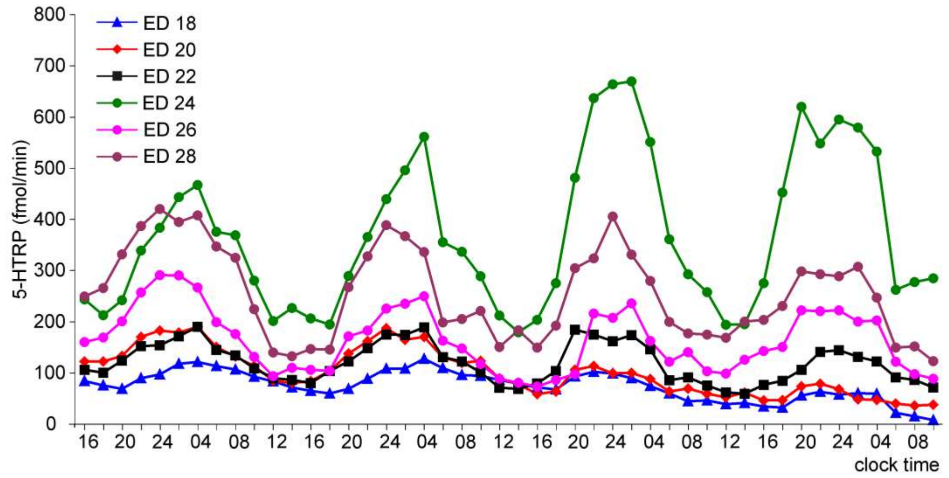


**Figure S1.** Comparison of 5-HTRP release from the pineal organs of ED 18 – ED 28 embryos incubated under a 12L:12D cycle.

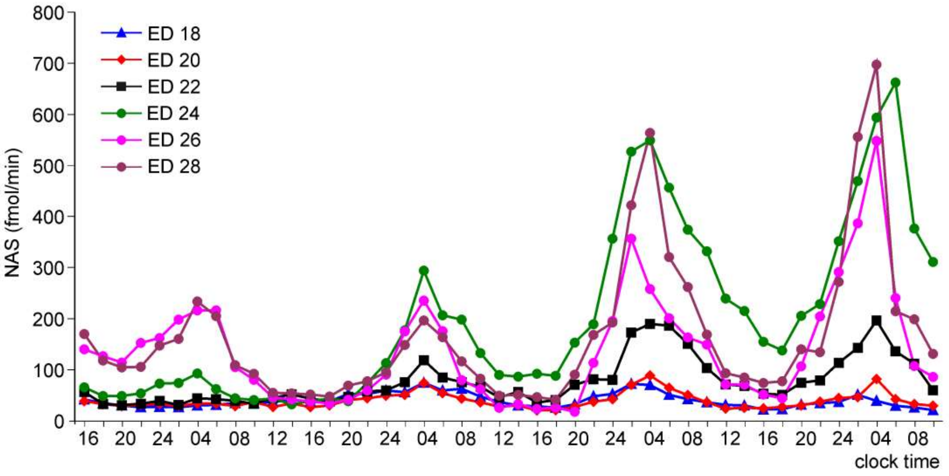


**Figure S2.** Comparison of NAS release from the pineal organs of ED 18 – ED 28 embryos incubated under a 12L:12D cycle.

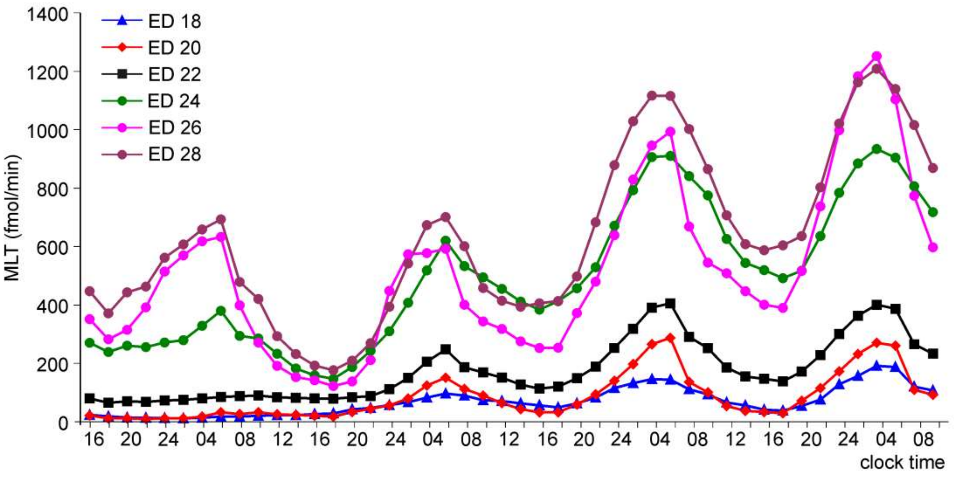


**Figure S3.** Comparison of MLT release from the pineal organs of ED 18 – ED 28 embryos incubated under a 12L:12D cycle.

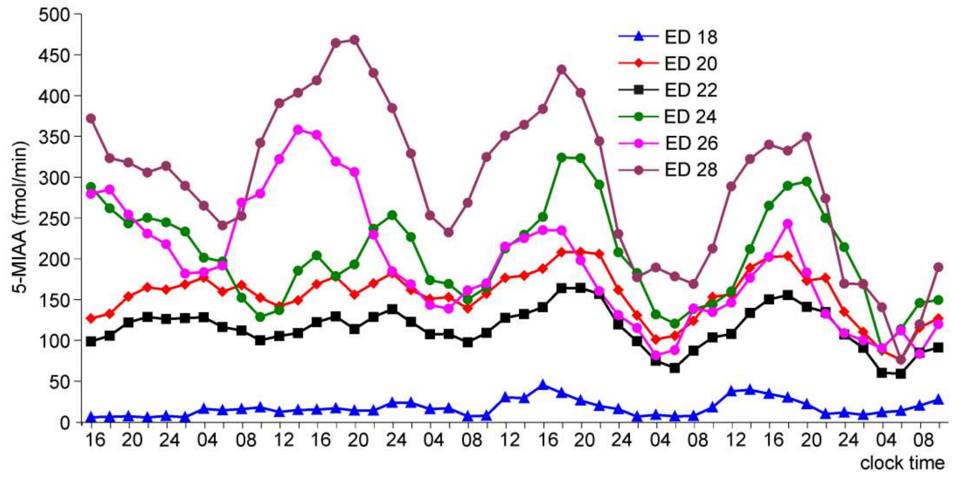


**Figure S4.** Comparison of 5-MIAA release from the pineal organs of ED 18 – ED 28 embryos incubated under a 12L:12D cycle.

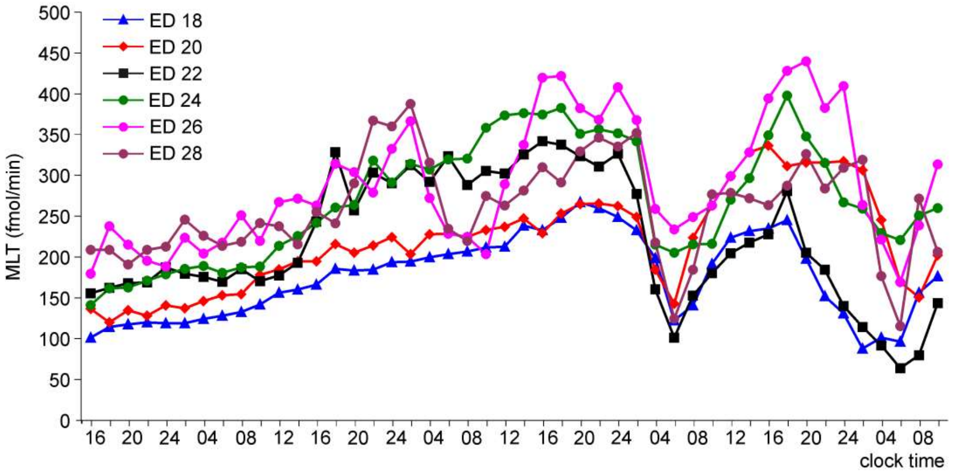


**Figure S5.** Comparison of 5-HTRP release from the pineal organs of ED 18 – ED 28 embryos incubated under a 12L:12D cycle and treated with NE during photophase.

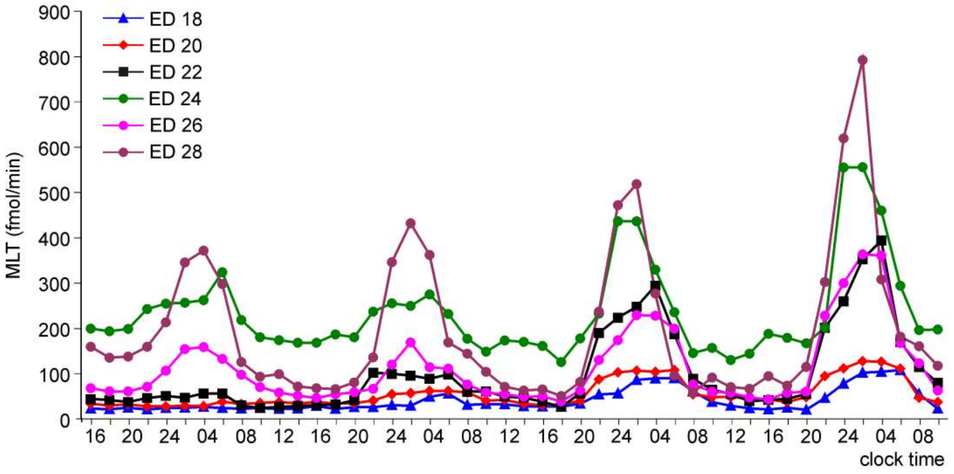


**Figure S6.** Comparison of NAS release from the pineal organs of ED 18 – ED 28 embryos incubated under a 12L:12D cycle and treated with NE during photophase.

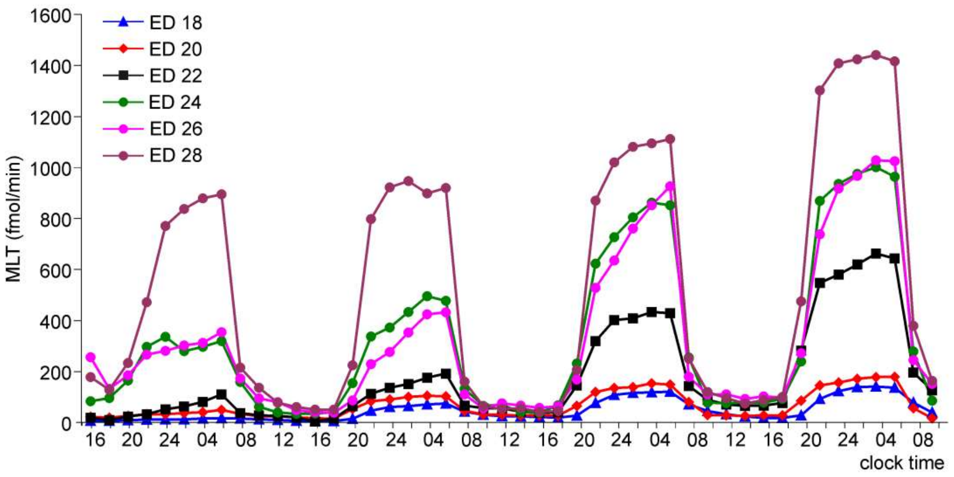


**Figure S7.** Comparison of MLT release from the pineal organs of ED 18 – ED 28 embryos incubated under a 12L:12D cycle and treated with NE during photophase.

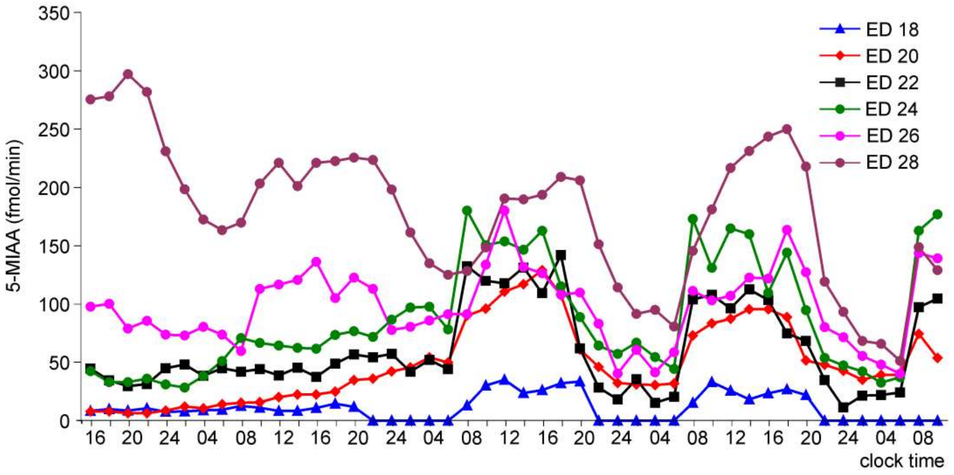


**Figure S8.** Comparison of 5-MIAA release from the pineal organs of ED 18 – ED 28 embryos incubated under a 12L:12D cycle and treated with NE during photophase.

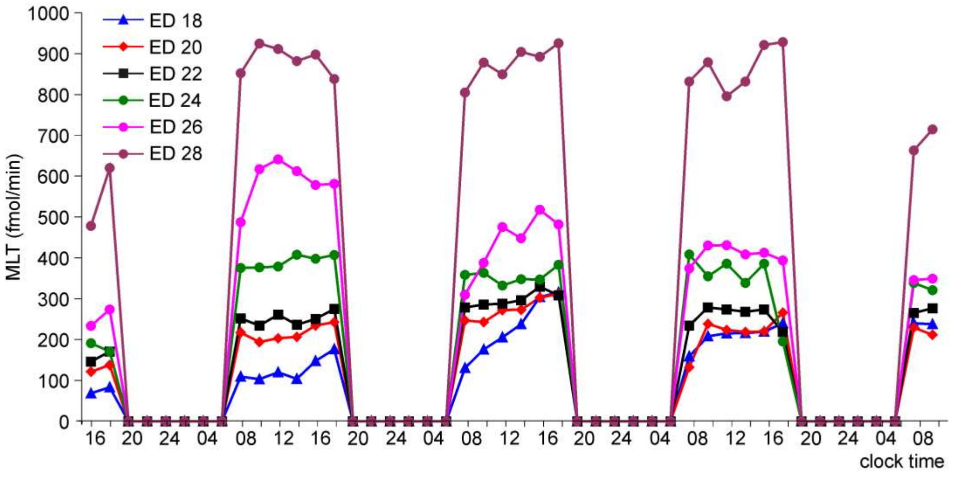


**Figure S9.** Comparison of 5-HT release from the pineal organs of ED 18 – ED 28 embryos incubated under a 12L:12D cycle and treated with NE during photophase.

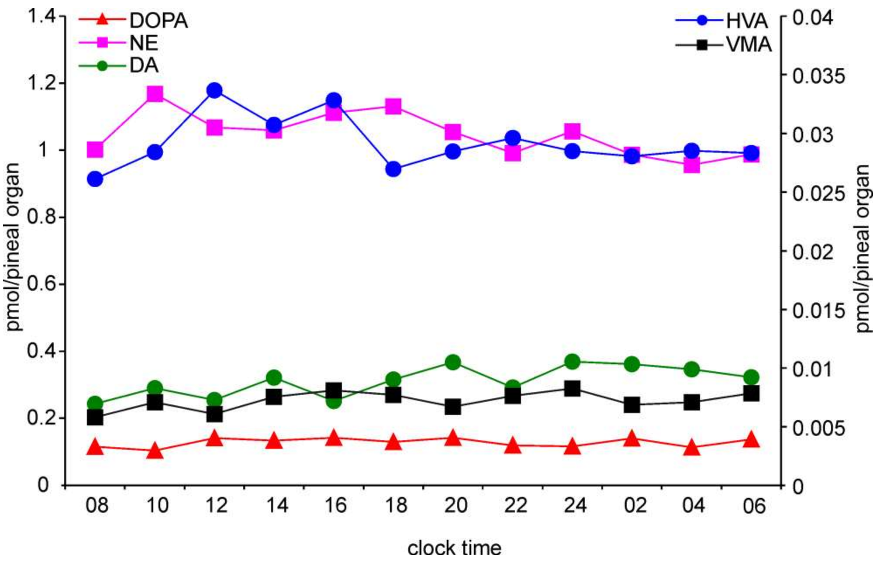


**Figure S10.** Content (means, n=5) of norepinephrine (NE), vanillylmandelic acid (VMA), dopamine (DA), homovanillic acid (HVA), and DOPA in the pineal organs of ED 24 embryos collected every 2-hrs. The means did not differ significantly between time-points.

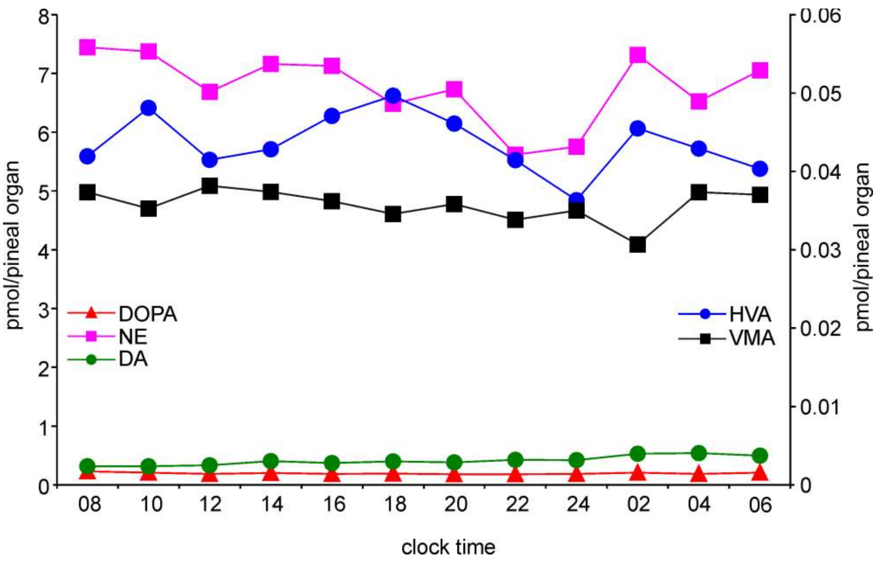


**Figure S11.** Content (means, n=5) of norepinephrine (NE), vanillylmandelic acid (VMA), dopamine (DA), homovanillic acid (HVA), and DOPA in the pineal organs of ED 26 embryos collected every 2-hrs. The means didnot differ significantly between time-points.

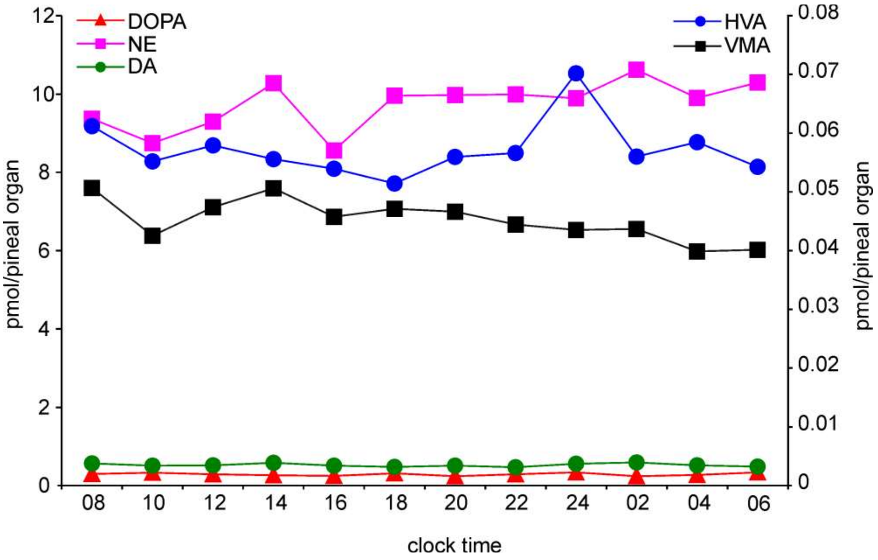


**Figure S12.** Content (means, n=5) of norepinephrine (NE), vanillylmandelic acid (VMA), dopamine (DA), homovanillic acid (HVA), and DOPA in the pineal organs of ED 28 embryos collected every 2-hrs. The means did not differ significantly between time-points.
